# ‘Misfit’ and ‘jack of all trades’: A qualitative exploration of the structure and functions of a network administrative organisation in Ontario, Canada

**DOI:** 10.1177/13558196251330524

**Published:** 2025-03-29

**Authors:** Jenna M Evans, Elana Commisso, Meena Andiappan

**Affiliations:** 1 Associate Professor, DeGroote School of Business, 120456 McMaster University, Hamilton, ON, Canada; 2 Research Associate, DeGroote School of Business, 120456 McMaster University, Hamilton, ON, Canada; 3 Associate Professor, DeGroote School of Business, 120456 McMaster University, Hamilton, ON, Canada

**Keywords:** network governance, network administrative organisation, performance management

## Abstract

**Objectives:**

Larger, more complex inter-organisational networks with strong, centralised governance structures, often in the form of a network administrative organisation (NAO), have developed in recent years in response to wicked health and social problems. Set in Ontario, Canada, this study explored how NAOs and networks are structured, how they function, and how they evolve.

**Methods:**

We conducted a case study of a NAO and network consisting of 40 member networks in the province of Ontario, Canada. We analysed secondary sources, including policy documents, legislation, contracts, websites, and existing qualitative data.

**Results:**

The NAO and member networks developed in tandem and dialectically. They ultimately took on a form that defies categorisation within the existing literature due to their structure as a ‘network of member networks’ and by acting simultaneously as a policy network, service delivery coordination network, and governance network, by executing numerous complex mandates and functions in service of multiple stakeholders, and by exemplifying both high control and high collaboration.

**Conclusions:**

We classified the NAO and its network as a ‘misfit’ and ‘jack of all trades’. These features may help explain its perceived effectiveness. The complexity and hybrid nature of the NAO and network may position it to best address multifaceted health care problems.

## Introduction

The implementation of inter-organisational networks in health care has raised questions regarding how to effectively govern them.^1^ These networks consist of three or more autonomous organisations working together to achieve a common purpose.^2,3^ Because member organisations are autonomous, hierarchical governance mechanisms traditionally used within organisations are absent or weak.^2^ Formal network governance structures are thus required to coordinate and control joint action in pursuit of network members’ shared goals.^2–5^ Networks may be governed collectively by the member organisations themselves, by a single member organisation that takes on a lead role, or by an external organisation known as a network administrative organisation (NAO).^3^

Centralised, externally brokered network governance through a NAO is a model increasingly used to oversee inter-organisational networks that seek to address multifaceted health and social issues such as antibiotic resistance,^6^ smoking cessation,^7^ and organ donation.^8^ These larger, more complex networks require a strong central governing body to ensure network goal achievement and accountability to the public.^9^ Theory and evidence suggest that the NAO is the most effective network governance model for large, complex networks.^10–13^

Networks are typically classified into one of three categories: policy, service delivery, or governance.^14^ Network governance forms are often conceptualised as either collaborative peer relationships through shared or lead organisation models, or as controlling hierarchical relationships through NAO models.^3,4^ However, the structure and roles of some NAOs and networks are more complex than these classifications suggest; NAOs and networks that seek to tackle ‘wicked’ health and social problems can be simultaneously involved in policy, service delivery and governance, and they can emphasise both collaboration and control. The structure, functions, and evolution of these types of NAOs is not well understood.

This study contributes to filling this knowledge gap by examining the structure, functions, and evolution of Cancer Care Ontario (CCO), a NAO in the province of Ontario, Canada. At the time of writing, CCO oversaw 40 integrated service delivery networks, which together form a ‘network of member networks’, a unique type of network in which each of the 40 member networks consists of multiple member organisations. CCO performs several functions, including administering funding, conducting policy development and implementation, coordinating service delivery, evaluating performance, and providing accountability oversight to the public for quality of care. The CCO network is both highly centralised and highly collaborative and characterised by many members, diverse stakeholders, functional complexity, and elaborate governance mechanisms. CCO has previously been described as high-performing and internationally renowned, and it was proposed as a model for the management of other chronic diseases.^15–17^ For example one study of CCO presented empirical evidence for sustained improvement in 25 out of 28 performance indicators over a 15-year period.^16^ The CCO model has since been replicated in Ontario’s renal system and used to help reform Ontario’s mental health and addictions system (please see Online Supplement for additional references).

### Theoretical background

Inter-organisational networks in public administration are typically categorised based on their primary function, whether it is to formulate and implement policy (policy networks), deliver services or coordinate their delivery (service delivery networks), or solve a societal problem and generate public value (governance networks).^14^ Regardless of the type, all networks require mechanisms for governing, that is, for coordinating and controlling joint action towards network members’ shared goals.^2–5^ Modes of network governance can be distinguished by the extent to which governance is distributed or centralised and internally or externally brokered.^3^ Based on these axes, networks are governed collectively by member organisations (distributed and internally brokered), by one member organisation that takes on a lead role (centralised and internally brokered), or by an external organisation known as a NAO (centralised and externally brokered).^3^

NAOs are a form of network governance in which a separate administrative entity is set up specifically to govern the network and its activities. A NAO may be created voluntarily by its member organisations or mandated by a third party through legislation or contract. NAOs range from a single individual acting as a facilitator to a formal organisation with employees and board members. It may be non-profit, for-profit or a government entity. It oversees a network, but is not a member itself, and usually operates separately from the organisations it oversees, serving as a centralised location to conduct network activities. NAOs normally focus their efforts on building and directing the network, allocating funds and other resources, supporting members to achieve network goals, and evaluating network effectiveness.^3,18^

NAOs have been described as the “more organisationally sophisticated and demanding mode of network governance”,^13^ (p. 17) but also the more effective mode under certain conditions.^11,19^ They are considered optimal for managing a high number of network members and harnessing network-level competencies to support goal achievement,^3^ provided the NAO is widely trusted and respected by network members.^20^ Compared to other governance forms, NAOs are also considered best suited to balance tensions inherent in network governance, including balancing efficient operation with inclusive decision-making; external legitimacy to non-member stakeholders with internal legitimacy among participants; and unity needed for coordination with diversity needed for innovation.^3,12^ NAOs have the ability to balance these tensions simultaneously or sequentially because they have a centralised structure for handling administrative burdens and serving as the ‘face’ of the network as well as representative participatory processes for network members to provide input and make decisions.^3,21^

Empirical studies of NAOs have examined the evolution of NAOs and networks,^5,7,22,23^ different NAO and network structural configurations,^21,24^ how they foster buy-in, participation, and consensus-building,^25^ and how they manage power and conflict.^5,23^ Most studies of NAOs have focused on small-to medium-sized NAOs in terms of number of staff (fewer than 20)^5,12^ and number of member organisations (<30).^5,12,23^ Furthermore, depending on their goals, networks engage in a range of activities from simply sharing information at one end of the continuum to joint production at the other, with those moving towards the latter more likely to be large and complex due to the increasing interdependencies and need for collaboration among network members.^26^ More complex NAOs are associated with rule-enforcing and member-sanctioning functions, which require sufficient operational capacity to execute.^24^ There is a lack of understanding of the structures, functions, and evolution of larger and more complex NAOs, in particular regarding how they balance multiple aims and functions, and how they overcome tensions between collaborating with and controlling network members.

In this study we used CCO as a case to examine the key structural, functional, and evolutionary features of the NAO that enable it to fulfill multiple mandates (including policy development, service delivery coordination, and governance oversight) across 40 networks. Our findings can inform theory development on complex NAOs and networks and enable policymakers, funders, and managers to establish network governance models capable of addressing challenging health and social problems.

## Methods

### Conceptualising Cancer Care Ontario as a network administrative organisation

There is no single common term to describe a NAO-type organisation; others have used descriptors such as ‘agency’, ‘body’, ‘office’, ‘council’, ‘central secretariat’, ‘authority’, and ‘platform’.^22–24^ There is also variation in NAO structures and functions,^12,24^ which makes it challenging to clearly define a NAO and distinguish it from similar organisational forms, such as shared services organisations.

We classified CCO as a NAO based on alignment of its purpose, structure, and functions with definitions and descriptions of NAOs in the literature. CCO is an organisation external to the network that governs the network and its activities.^3^ Like other NAOs, CCO was mandated by the government to address a social or health problem, has multi-regional members, and fulfills a range of functions ascribed to NAOs in the literature, including directing and coordinating the network, allocating funds to members, issuing contracts to members to deliver services to the public and holding members accountable for service performance, supporting members to achieve network goals, and evaluating network effectiveness.^3,5,7,18,23,25^ We note that CCO has selected features in common with shared service organisations, which centralise non-strategic functions such as information technology and human resources for multiple intra-organisational business units or across multiple organisations. However, we concluded that CCO’s network structure and functions go beyond a shared services approach, and are best described as a NAO.^27^

### Methodological approach

We used a qualitative case study approach, drawing on existing qualitative data. We first developed a historical timeline of the formation and evolution of CCO using publicly available reports, legislation, web sites, news articles, and published manuscripts (please see Online Supplement 1), as well as internal CCO documents, including network contracts, policy documents, and network performance reports. We used these sources to extract information on CCO’s roles and functions, which we verified with a manager at CCO. We supplemented the information using qualitative data generated as part of a large multiyear grant from Canada’s Social Sciences and Humanities Research Council, which included 243 semi-structured interviews with leaders, care providers, and staff from CCO and the member networks between 2017 and 2020. Interviews included data on participant perceptions of CCO’s structure, roles, and functions (see Online Supplement Table S1 for an overview of interview participants).

Interview participants were recruited through purposeful sampling with the support of CCO and network executives who provided a list of eligible administrative and clinical representatives. Interviews with CCO representatives were primarily conducted in person, while those with network representatives were primarily done by phone due to geographic dispersion. All participants provided verbal informed consent to participate in an interview and all were asked the same set of questions. Relevant to this study were the first few questions which probed on participant perceptions of CCO’s roles, how it functions, its relationship with the networks, and strengths and weaknesses of its approach to performance management. The interviews were digitally recorded, transcribed verbatim, and inductively coded using NVivo software. For the purposes of this paper, we reviewed node reports for codes related to network member perceptions of CCO and followed thematic analysis methodology to identify recurrent patterns in the data. The interview studies received ethics approvals from the University of Toronto Research Ethics Board (protocol #00036408) and McMaster Research Ethics Board (MREB #2405).

## Results

### Formation of CCO as a network administrative organisation

As a crown agency, CCO was owned by the Government of Ontario but operated at arm’s length from the government. CCO was classified as an Operational Service Agency (OSA) of the Ontario Government, that is, a board-governed provincial agency that delivers goods and services to the public usually with no, or only minimal, fees. OSAs like CCO are created to provide independent expert advice, deliver a service using specialised expertise or capabilities, and ensure public confidence in the impartiality of decisions or actions.

CCO was established in 1943 by law (Cancer Act) as the Ontario Cancer Treatment and Research Foundation (OCTRF), with the mandate to centralise cancer care in Ontario by creating and managing a programme of cancer diagnosis, treatment, and research. The OCTRF opened eight Regional Cancer Centres (RCCs) across the province, which brought together many disciplines of cancer care under one roof. However, most cancer services continued to be provided outside of the RCCs; therefore, cancer services remained fragmented with varying degrees of centralisation and decentralisation.

In 1997, OCTRF became Cancer Care Ontario (CCO) and was given a broader mandate, including coordinating and improving all cancer treatment services and providing the Government of Ontario with strategic direction on cancer care. However, there were growing public concerns about waiting lists and the ability of the system to meet the need for cancer services, resulting in a restructuring of the cancer system in 2004. CCO was given a stronger, more comprehensive mandate to improve performance by driving quality, accountability, and innovation in all cancer-related services. It became the funder of a broader range of cancer services, with direct service delivery delegated to 11 RCCs, which were formally integrated with their host hospitals to form Integrated Cancer Programs (ICPs). This process was voluntary. The host hospitals became employers and contract agents for the ICP, with a Regional Vice President (RVPs) appointed for each. Each RVP has dual accountability to the CEOs of both CCO and their host hospital. Fourteen Regional Cancer Programs were established to link ICPs with external organisations and professionals involved in cancer prevention and care in their region to create 14 integrated service delivery networks.

#### CCO clinical leadership structure

CCO’s clinical leadership structure comprises Regional and Provincial Clinical Leads who are based at various hospitals in Ontario. Regional Clinical Leads bring their respective regional perspectives to inform CCO’s provincial quality agenda; they also serve as champions for implementation of quality initiatives within their hospitals and regions. Provincial Clinical Leads provide oversight and support Regional Clinical Leads. Together, Regional and Provincial Clinical Leads represent a range of health care services and specialities along the continuum of care (e.g., prevention, screening, imaging, surgery, psychosocial care) and specific disease sites (e.g., breast, gynaecology, lung, head and neck). All Provincial Clinical Leads and most Regional Clinical Leads are paid by CCO for 1–2 days per week to participate in strategic planning, indicator selection and target-setting, guideline development, and implementation of quality improvement initiatives.

#### CCO board structure

CCO has a provincially appointed board of external professionals and experts. The board is responsible for ensuring CCO fulfills its mandate and holds CCO accountable to the Ontario government for its performance and use of public funds. The board is complemented by two structures that enable network members to govern the network through representation and deliberation. The Clinical Council brings together Provincial and Regional Clinical Leads to develop clinical policies and quality improvement priorities for the network. The Provincial Leadership Council brings together the RVPs from member networks and the CCO executive team to plan and implement network strategies, facilitate network coordination, and evaluate network effectiveness.

#### Ontario Renal Network

In 2009, the Ontario Renal Network (ORN) was established to manage the delivery of chronic kidney disease (CKD) services under the auspices of CCO. The aim was to replicate the province-wide approach to managing and improving cancer care for CKD services. The acronym CCO was used to refer to the organisation as a whole, including the cancer and renal branches, whereas *Cancer Care Ontario* was used to refer to the cancer branch of the organisation. Like Regional Cancer Programs, Regional Renal Programs bring together multiple hospitals, including the host hospital which serves as the hub and liaison with CCO, and other service providers, to form 27 integrated service delivery networks. Regional Directors embedded in host hospitals are employees of, and therefore accountable to, both their host hospital and CCO. Together with Regional Clinical Leads they liaise between their hospital/network and CCO and support the design and implementation of CCO initiatives to improve renal care.

**Figure 1.**
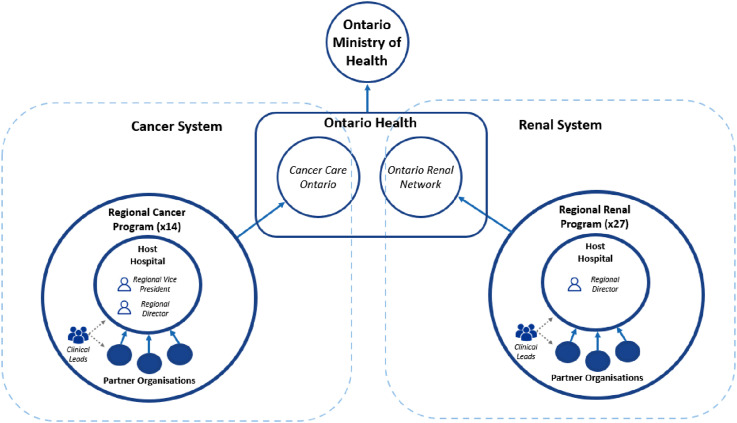
Structure and accountability relationships in Ontario’s cancer and renal care systems. Note. Arrows denote accountability relationships.

In 2019, CCO became part of ‘Ontario Health’ through a merger of 20 agencies in Ontario following the Connecting Care Act, which aimed to create a more patient-centered, integrated health system. CCO principally retained its structures and functions; the only exception was the creation of a single board for all merged agencies. CCO and the ORN operate as independent branches within Ontario Health. Figure 1 illustrates the current CCO network structure and accountability relationships. In what follows we use ‘CCO’ to refer to both the cancer and renal branches.

### Network and NAO structure and functions

**Figure 2.**
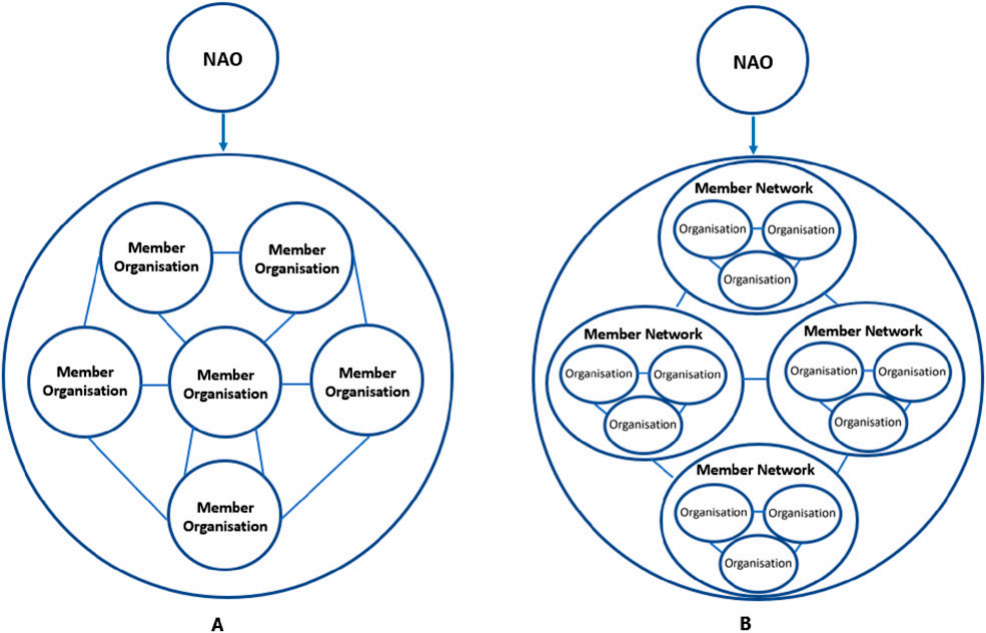
Forms of network administrative organisation and network structures. (a) Inter-organisational network with a NAO, (b) Network of member networks with a NAO

Ontario’s 14 cancer networks collectively form a cancer system network, and the 27 renal networks collectively form a renal system network, creating a unique ‘network of member networks’. This means that CCO oversees multiple member networks, each consisting of multiple organisations (Figure 2, panel B).

CCO’s mandate is to improve the performance of the cancer and renal systems by driving quality, accountability, innovation, and value in all services. This broad mandate contributes to CCO’s wide range of functions, spanning all three of the core ‘trades’ of networks, that is, policy development and implementation, service delivery and coordination, and governance and accountability for performance (Box 1).

**Box 1.** Functions of cancer care Ontario.

**Policy development and implementation**
1. Advises the Ontario government on cancer and renal care
2. Leads the development of multi-year cancer and renal plans that outline strategic objectives to guide system improvement
3. Establishes evidence-based guidelines and standards for cancer and renal care
**Service delivery and coordination**
4. Allocates and distributes over $1.5 billion every year for cancer and renal services through funding and service agreements with hospitals and other providers
5. Manages and coordinates networks of regional cancer and renal programs across the province
6. Conducts service planning and modelling to estimate funding and resource needs province-wide for implementation of innovations
**Governance and accountability**
7. Develops and implements information systems to collect performance data on cancer and renal services
8. Collects, analyses, and reports data on performance of cancer and renal services
9. Manages the performance of regional cancer and renal programmes through fiscal and performance-based agreements for service volumes, data submission, and implementation of quality initiatives
10. Engages patients and families in the design, delivery, and evaluation of cancer and renal services

CCO’s ‘trades’ are supported by hierarchical, contractual, and relational governance mechanisms, which are embedded in CCO’s performance management system. The performance management system comprises numerous inter-related tools and processes that mandate requirements, share performance data, stimulate performance dialogue and problem-solving between CCO and the networks, and establish rewards and sanctions for performance. For example, the performance management system includes funding contracts that set out performance expectations and deliverables, with funding at risk for non-compliance; a scorecard with indicators, targets, and network rankings; web-based access to performance data; quarterly performance review reports and meetings; annual performance recognition certificates; an escalation process for poor or declining performance that involves a formal letter from CCO to the hospital and network and the development and execution of an improvement action plan; and public reporting of select indicators.

### Stakeholder perceptions of CCO as a NAO

Network members interviewed for this study generally recognised CCO’s diverse mandates and they viewed performance management as a core function through which CCO pursues its mandates. Participants reported experiences of both top-down control and bottom-up collaboration in their interactions with CCO.

NAO and network representatives emphasised CCO’s role as a government advisor that oversees the cancer and renal care systems with the aim of *“driving quality, accountability, and innovation”* (CCO P014). CCO was described as *“the ultimate steward”* of funds, strategic directions, and performance (Clinical Lead P054), as a *“quality performance setting organisation”* (Clinical Lead P046), and as a *“provincial repository”* of performance data and best practices (Network P060). Performance management was thus perceived to be a primary function of CCO by network members, one that supports the NAO’s policy, service coordination, and governance mandates, with one study participant describing performance management as *“fundamental to our whole purpose of existence”* (CCO P028). CCO was further viewed as a *“leader that moves us in the direction we want to be”* (Network P057) and an entity that *“guides the ship in newer directions”* (Clinical Lead P046) using policy, strategic plans, funding, capacity building, and rewards and sanctions (*“carrots and sticks”*; Clinical Lead P053; CCO P023).

Participants identified two fundamental enablers of CCO’s ability to steer: funding and the leadership structure. The contracts between CCO and the networks that tie performance requirements to funding were repeatedly identified as a “*huge* strength” by CCO and network representatives (CCO, P002; Network P060): *“If we didn’t have the funding lever and we weren’t able to hold them accountable, I don’t think we would be as successful”* (CCO P023). The leadership model, which embeds administrative and clinical leaders in the networks who are accountable to CCO was identified as a *“core strength”* (Network P060); the leadership structure was seen as fostering *“strong interpersonal relationships”* (Network P058), *“a sense of ownership”* (CCO P023) and *“a web of influence and accountability”* (CCO, P002) that enable CCO to fulfill its mandates. These descriptors of CCO’s steering capabilities suggest that this network of member networks is highly centralised. At the same time, CCO’s internal structure is reliant on departmental divisions that enable some decentralisation of decision-making within the organisation and high NAO competency-building in specialised areas (e.g., performance management unit, contracts unit, standards unit).

While the network as a whole appears highly centralised, CCO was also described as a *“partner”*, *“supporter”*, *“facilitator”*, and *“enabler”* of the networks in improving quality (CCO P004; CCO P017; Clinical Lead P051). Study participants’ accounts point to an organisation that manages its many diverse members and stakeholders through a highly collaborative approach even in the context of a strong top-down governance structure. The centralised, top-down structure appears flexible, not rigid, with strong feedback loops that inform change.

*The performance process includes the clinicians. It’s not just CCO. CCO facilitates it and coordinates it, but the [networks] are very much involved. *(CCO P017)

*[T]here are no marching orders that tell you, you must start on your left foot. That’s up to us locally, how are we going to achieve this...We figure out what fits best because we are the experts on our context and the services we provide.* (Network P049)

The balance between centralised top-down control and bottom-up collaborative engagement seems to be at the core of what makes CCO as a network of member networks function effectively.^15^

*We adopt a very collaborative approach [with the networks] but collaboration only works when everybody agrees. When there is disagreement, we have moral persuasion, and they always have to believe you have a big stick in the drawer which hopefully you’ll never have to use.* (Clinical Lead P033)

*There are times when CCO is a top-down driver of change and there are times when it’s grassroots…allowing the individual centres and programs to come together to devise a strategy on how to achieve [a goal].* (Network P041)

The dualism of centralisation and collaboration, while productive, also appeared to contribute to tensions. For example, there were accounts of confusion regarding the scope of CCO’s role versus the networks, with one participant asking *“how far should they [CCO] reach into the networks?”* (Network P032). We heard from participants who wanted CCO to step back while there were others who wanted CCO to offer more, particularly regarding support for quality improvement. There were also concerns about the nature and number of strategic priorities; performance indicator selection, measurement, data quality, and target-setting; the burden associated with data collection and quality improvement initiatives; use of the funding clawback as a consequence for non-compliance; and the influence of local contextual differences on performance. Overall, participants rarely questioned the existence, structure, or core functions of CCO, but rather centered their critiques on what participants called the *“nitty gritty”* (Clinical Lead P036) or *“fine points”* (Network P060), highlighting that the main reservations related to how CCO operationalises its role and functions.

## Discussion

This study explored CCO as a case of a NAO and network that invites us to rethink existing descriptions of NAOs. The network is structured as a ‘network of member networks’ and acts simultaneously as a policy network, service delivery coordination network, and governance network. As the NAO of this ‘network of member networks’ with a wide range of mandates and functions, CCO can thus be characterised as a ‘misfit’ and ‘jack of all trades’ within the literature on network governance and NAOs. The CCO case suggests the need and opportunity to expand current conceptualisations of NAOs and the networks they oversee to include more complex and dynamic configurations.

Our study also provides new insights into how networks of member networks are governed. NAOs overseeing complex networks of member networks need to govern in an agile and multifaceted way. Thus, they require certain structural features and functional abilities that allow for such behaviours. In the case of CCO, key features include funding the networks and financial incentives to promote compliance with NAO requirements, leaders with dual accountability to their network and the NAO, and a robust performance management system that uses both control and collaboration to achieve the NAO’s policy, service coordination, and governance mandates.

Available evidence suggests that CCO is high-performing and internationally renowned.^15–17^ Our findings therefore suggest that complex and hybrid NAO and network forms can be more effective than traditional structures in addressing complex problems. NAOs tend to be the most effective network governance model for large, complex networks,^10–12^ and others have also noted that hybrid network governance approaches that embrace both control and collaboration can maximise performance.^15,28–30^

Our findings suggest that high-performing NAOs develop in a dialectical relationship to their network. We note that the evolution of CCO was largely driven by fragmented and untimely cancer care and in response, CCO’s mandate was progressively expanded and modified to deliver more coordinated and timely care to patients, thereby moving CCO from a service delivery provider to a NAO. In parallel, the cancer system was fundamentally restructured involving both regulatory (legal changes) and voluntary measures (hospital restructuring). As such, the networks and the NAO developed in tandem and iteratively, impacting and informing one another and the broader system within which they operate, to maintain quality and accountability to multiple stakeholders and the public at large.

### Limitations

We classified CCO as a NAO, but, due to its complexity, it is possible that a different label may be required to best describe its organisational form or that it is a specific sub-type of NAO. Our study can help inform future empirical research to better distinguish similar organisational types and develop a more nuanced typology of NAOs to enhance conceptual clarity and boundaries. Further, although we used multiple sources of data, drawing on various historical sources and an existing qualitative dataset, there is a need for more detailed qualitative research on stakeholder perspectives and experiences to explore issues around how stakeholders shape the development and evolution of NAO and network structures and functions, and whether network aims and functions as codified in policy or legislation are realised on the ground. It was beyond the scope of this paper to conduct comparative case study work, but we believe that such research would help strengthen the evidence base around complex network forms. Finally, our study points to the need for further work on the implications of network governance structures that operate between the government and on-the-ground organisations and networks, including their evolution. For example, can the structures and functions we identified be put in place at the outset of network formation or do they necessarily develop as the network matures as they did in the CCO case?

### Implications for policy and practice

Policymakers and managers are facing an increasing number of health and social issues that are multifaceted, urgent, and uncertain, such as pandemics and climate change. Larger and more complex inter-organisational networks provide a means to better address such challenges. Our study suggests that emerging networks and network governance forms may not fit neatly into the categories outlined in the literature; rather they may span them, taking on complex, hybrid forms to tackle challenging problems more effectively. Findings from this study may inform the (re)design of networks and network governance structures in other jurisdictions and for other problems in which public intervention is necessary. A ‘network of member networks’ structure, which combines and leverages the benefits of both small network and large network membership might provide a suitable model. NAOs could be given greater control over funding and resource distribution, and they may also explicitly combine policy, governance, and service delivery or coordination mandates into the network, thereby maximising coordination and synergy across activities that are traditionally led by discrete organisations or networks. Strong centralised and control-oriented governance mechanisms could be complemented by strong collaborative governance mechanisms and feedback loops.^15^ Finally, our findings suggest that as NAO and network design evolve, this will necessitate changes over time to meet new stakeholder needs and address environmental shifts.

## Supplemental Material

Supplemental Material - ‘Misfit’ and ‘jack of all trades’: A qualitative exploration of the structure and functions of a network administrative organisation in Ontario, Canada Supplemental Material for ‘Misfit’ and ‘jack of all trades’: A qualitative exploration of the structure and functions of a network administrative organisation in Ontario, Canada by Jenna M Evans, Elana Commisso and Meena Andiappan in Journal of Health Services Research & Policy
