# Supplementary material for: ‘Misfit’ and ‘jack of all trades’: A qualitative exploration of the structure and functions of a network administrative organisation in Ontario, Canada
Source: J Health Serv Res Policy. 2025 Mar 29;30(3):152–60. doi: 10.1177/13558196251330524 (PMC12134403; doi:10.1177/13558196251330524)
Supplement: Supplemental Material - ‘Misfit’ and ‘jack of all trades’: A qualitative exploration of the structure and functions of a network administrative organisation in Ontario, Canada [file sj-pdf-1-hsr-10.1177_13558196251330524.pdf]

Online Supplement 1: Cancer Care Ontario: information sources

The following references were used to develop a historical timeline of the formation and evolution of CCO as well as a description of its current structure and functions.

1. Bell B. Don't harm Cancer Care Ontario while restructuring health agencies [Internet]. thestar.com. 2019 [cited 2021 October 25]. Available from: https://www.thestar.com/opinion/contributors/2019/01/17/dont-harm-cancer-care-ontario-while-restructuring-health-agencies.html
2. Burak R. Report of the special advisor on agencies [Internet]. 2010 [cited 2023 May 5]. Available from: https://docs.ontario.ca/documents/2031/burak-report-on-agencies.pdf.
3. Cancer Care Ontario (CCO). Annual Report 2016/17 [Internet]. 2017 [cited 2023 May 5]. Available from: https://www.ccohealth.ca/sites/CCOHealth/files/assets/CCO_AnnualReport2016-17_ENGLISH_DIGITAL.pdf
4. Denis J-L, Usher S, Preval J. Health reforms and policy capacity: the Canadian experience. Policy and Society. 2022;42(1):64-89.
5. Duvalko KM, Sherar M, Sawka C. Creating a System for Performance Improvement in Cancer Care: Cancer Care Ontario's Clinical Governance Framework. Cancer Control. 2009;16(4):293–302.
6. Frketich J, LaFleche G. Super agency brings end to independent Cancer Care Ontario [Internet]. 2019 [cited December 30, 2022] *Hamilton Spectator*. Available from: https://www.thespec.com/news-story/9510881-super-agency-brings-end-to-independent-cancer-care-ontario/
7. Legislative Assembly of Ontario. The People's Health Care Act, 2019 (Transcript from April 2, 2019 meeting of the Standing Committee on Social Policy) [Internet]. 2019 [cited 2023 December 26]. Available from: https://www.ola.org/en/legislative-business/committees/social-policy/parliament-42/transcripts/committee-transcript-2019-apr-02

8. Ministry of Health. Roadmap to wellness: A plan to build Ontario's mental health and addictions system [Internet]. 2022 [cited 2023 December 26]. Available from: https://www.ontario.ca/page/roadmap-wellness-plan-build-ontarios-mental-health-and-addictions-system
9. Ministry of Health. Connected Care update – November 13, 2019 [Internet]. 2023 [cited 2023 December 26]. Available from: https://www.ontario.ca/document/connected-care-updates/connected-care-update-november-13-2019
10. Rachini M. Teen mental health support can learn from cancer care in Ontario, psychologist says [Internet]. 2023 Sep 21 [cited 2023 December 26]. Available from: https://www.cbc.ca/radio/thecurrent/cancer-care-ontario-teen-mental-health-1.6972977
11. RISE (Rapid Improvement Support and Exchange). RISE brief 29: Examining intersections between Ontario Health Teams and specialty service lines [Internet]. 2022 [cited 2023 December 26]. Available from: https://www.mcmasterforum.org/docs/default-source/rise-docs/rise-briefs/rb29_intersections-specialty-service-lines.pdf?sfvrsn=e5719c78_12
12. Sawka C, Ross J, Srigley J, Irish J. The Crucial Role of Clinician Engagement in System-Wide Quality Improvement: The Cancer Care Ontario Experience. Healthcare Quarterly. 2012;15(sp):38–41.
13. Sullivan T, Dobrow M, Thompson L, Hudson A. Reconstructing Cancer Services in Ontario. HealthcarePapers. 2004;5(1):69–80.
14. Thompson L, Martin M. Integration of Cancer Services in Ontario: The Story of Getting It Done. Healthcare Quarterly. 2004;7(3):42–8.
15. Woodward G, Iverson A, Harvey R, Blake P. Implementation of an Agency to Improve Chronic Kidney Disease Care in Ontario: Lessons Learned by the Ontario Renal Network. Healthcare Quarterly. 2014;17(SP):44–7.

Table S1. Interview participant characteristics (n=243)*

**Demographics**	**Percentage**
Network administrative organisation's leaders and staff (n=78)	
Role	
Administrative	81% (63/78)
Clinical (i.e., provincial clinical leads)	19% (15/78)
Networks' leaders, clinicians, and staff (n=165)	
Type of network	
Cancer	55% (91/165)
Renal	45% (74/165)
Role	
Administrative	62% (102/165)
Clinical	38% (63/165)

*Some individuals were interviewed more than once at different time points, but are counted separately in the table
